# Last Advances in Silicon-Based Optical Biosensors

**DOI:** 10.3390/s16030285

**Published:** 2016-02-24

**Authors:** Adrián Fernández Gavela, Daniel Grajales García, Jhonattan C. Ramirez, Laura M. Lechuga

**Affiliations:** 1 Nanobiosensors and Bioanalytical Applications Group. Catalan Institute of Nanoscience and Nanotechnology (ICN2), CSIC, The Barcelona Institute of Science and Technology and CIBER-BBN, Campus UAB, Ed-ICN2, 08193 Bellaterra, Barcelona, Spain; adrian.fernandez@cin2.es (A.F.G.); daniel.grajales@cin2.es (D.G.C.); 2 School of Electrical and Computer Engineering (FEEC), University of Campinas (UNICAMP), 13083-970 Campinas, SP, Brazil; jcordoba@decom.fee.unicamp.br

**Keywords:** photonic biosensors, label-free detection, silicon photonics, waveguide devices

## Abstract

We review the most important achievements published in the last five years in the field of silicon-based optical biosensors. We focus specially on label-free optical biosensors and their implementation into lab-on-a-chip platforms, with an emphasis on developments demonstrating the capability of the devices for real bioanalytical applications. We report on novel transducers and materials, improvements of existing transducers, new and improved biofunctionalization procedures as well as the prospects for near future commercialization of these technologies.

## 1. Introduction

The need to monitor and detect biological elements, related to human and environment health, in a fast and reliable way, is one of the challenges faced by humanity at the dawn of the 21st century. Tests done nowadays in laboratories (such as ELISA, PCRs, cell cultures, *etc.*) are slow (requiring from several hours to days) and expensive. This can be either due to the pace of biology when using cell cultures or due to the use of fluorescent labels that make the detection process more complicated, including laborious sample preparations, the use of bulky equipment, slow data processing and the need of skilled personal. An ideal detection method should be simple, fast and direct (without labelling), with high sensitivity and selectivity, allowing multiple simultaneous detections at low cost by non-skilled personnel [1].

By definition, a biosensor is a device able to detect a specific analyte by converting the recognition by a biological receptor into an electrical signal that can be further processed and related to the concentration of the analyte [2]. Biosensors incorporate a biological element for sensing (DNA, RNA, antibody, enzyme, *etc.*), a physicochemical transduction element and a readout system for signal post-processing. Depending on the transduction principle biosensors are classified into electrochemical, mechanical, acoustic, calorimetric, optical, *etc.* Among these, optical biosensors offer the advantages of very high sensitivity, direct, real-time and label-free operation in addition to multiplexed capabilities [3,4].

Integrated optics (IO) is a technology born as part of photonics with the aim of building light wave circuits to make complex optical operations. IO is taking advantage of the silicon microelectronic industry via very standardized and well-known fabrication processes. By using IO it is possible to lower the production costs while adding mechanical stability and high levels of integration. In this sense, IO-based biosensor devices have a great potential for building devices capable of parallel operation making real-time-multiplexed recognition feasible [5,6]. Novel materials or fabrication process, using for example transparent glass substrates processed by femtosecond laser, could be a competitive alternative in a near future to improve integration and miniaturization of IO devices, but silicon is still the workhorse of the IO photonic research and industry. Devices can be cheaply built through mass production with industrial silicon technologies and they could become the core of portable laboratories. Such portable laboratories would have to deal with all the assay steps, normally carried out in a standard analytical chemistry laboratory, through the integration of all the functions in a single chip. These devices are called Lab-on-a-Chip (LOC) platforms [7]. They must be capable of delivering a readable signal easy to be interpreted by non-skilled personnel. They might include fluid handling, sample preparation (filtration, homogenization and dilution), analyte detection, transducer readout and signal processing [8].

**Figure 1:**
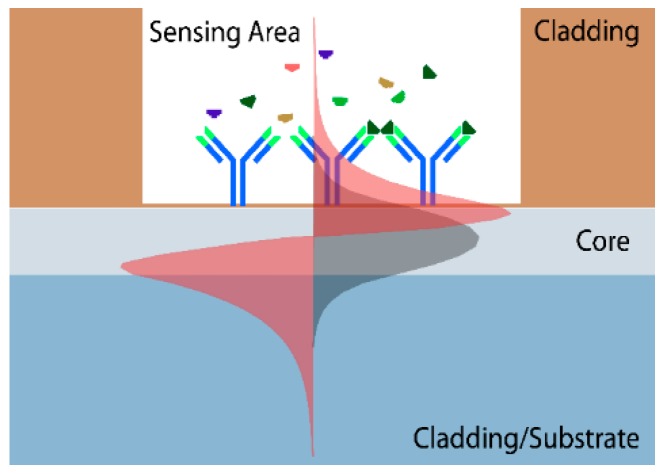
Evanescent wave detection principle.

The majority of optical label-free biosensors are based on the evanescent field detection principle: in a waveguide due to total internal reflection at the interfaces, light propagates through the core producing an evanescent wave at the substrate and cladding boundaries [9] (see Figure 1). If a sensing window is etched in the cladding, opening an access to the core surface, the behaviour of the guided light in the core is directly related to any perturbation taking place in the evanescent area over the surface.

In this review we try to summarize the publications over the last five years within the domain of IO label-free silicon biosensors. As previously mentioned, silicon is becoming the most employed material in IO, incorporating a whole family of related materials such as silicon nitride, polysilicon, and silicon dioxide. Furthermore, silicon combines well with different kinds of polymers [10,11] and emerging new nanomaterials such as graphene [12]. But many challenges still remain in the full system integration, industrialization and commercialization of complete LOC systems [13].

## 2. Optical Biosensors

### 2.1. Interferometric Biosensors

In an IO interferometric device, the incoming light beam from the source is divided in two beams that travel through different optical paths. For biosensor applications, one of the branches is used as a reference arm and the other as a sensing arm. The evanescent field of the propagated mode in the sensing arm interacts with the sample. The change introduced in the interference pattern, generated by the recombination of the propagated modes of each path, is proportional to any refractive index variation taking place in the evanescent field of the sensing arm. There are many different structures for interferometric devices [14], but we report here the most common configurations.

**Figure 2:**
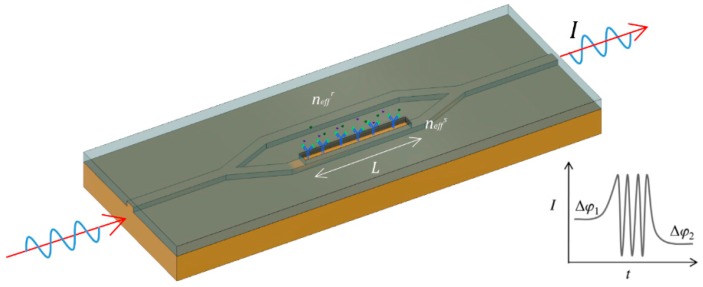
Standard integrated MZI and interferometric output.

In a standard integrated Mach-Zehnder Intereformeter (MZI), a laser beam is coupled in a single mode waveguide, normally operating in visible wavelengths. By using two integrated Y-junctions, the light is divided in the two arms described above and after certain distance both signals are recombined as Figure 2 shows. The output signal, with intensity, *I*, depends on the phase difference, *Δϕ(t)*, between the propagated beams for each branch, through the equations:

(1) I∝cos(Δφ(t))

(2) $$\Delta \varphi \left(t\right)=\frac{2\pi L}{\lambda }\left(n_{eff}^{s}-n_{eff}^{r}\right)$$

where *L* is the length of the sensing arm, *λ* the wavelength of the light, and $n_{eff}^{s}$ and $n_{eff}^{r}$ are the effective refractive indices of the guided modes in the sensing and reference arms, respectively.

The first integrated MZIs for biosensor applications based on silicon technology were reported in the 90s [15,16,17]. In order to improve the integration and sensitivity of these devices, new configurations were developed in the early 21st century [18,19,20] showing a sensitivity up to 1 × 10^−7^ RIU [21]. Silicon nitride (Si_3_N_4_) was employed as a core waveguide layer on the first integrated MZIs. Other structures based on silicon technology, like silicon wires, have also been developed over the years [22,23]. Other configurations, based on polymer [24] or glass [25], have also been successfully implemented, but the limit of detection (LOD), in the range of 10^−4^ RIU, generally remain worse compared to silicon-based MZIs.

**Figure 3:**
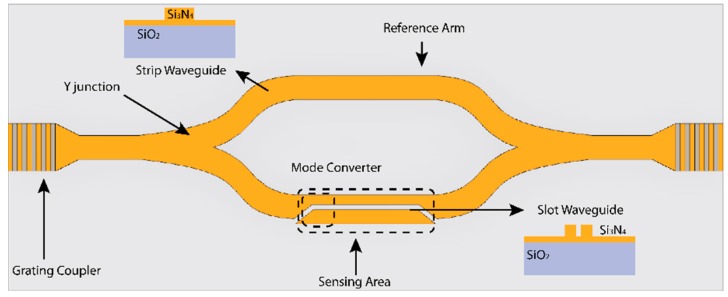
MZI with a slot waveguide as sensing arm, which is redrawn based on [30].

The trend over last few years has been to improve sensitivity, integration or cost-efficiency. In order to improve cost-efficiency of the fabrication process using Si-technology, alternatives to Si_3_N_4_ as a core layer have been developed such as SiO_x_N_y_, deposited using plasma-enhanced chemical vapour deposition (PECVD) [26,27]. Regarding integration, due to the low-cost of the light sources, photodetectors, and other optical components for visible wavelengths, make it easier to implement a portable LOC device. In addition, most of the biomolecules are non-absorbent in the visible spectra, avoiding any damage or light absorption. A key issue in the assembly of a truly portable LOC is light incoupling. Normally, this is solved by using grating couplers. For example, a LOC MZI biosensor using grating couplers and a low-cost diode as a source for multiplexed analysis has been proposed reaching a bulk sensitivity of 1.6 × 10^−7^ RIU [28], comparable with the most sensitive MZIs [2]. Using slot waveguides [29] for the MZI structure, integration and sensitivity can be improved. A MZI device with a 7 mm long slot waveguide (see Figure 3) as a sensing arm has achieved a sensitivity of 1864 π/RIU, and a detection limit of 5.4 × 10^−6^ RIU [30]. In order to integrate this configuration in a LOC device, a multiplexed system has been proposed for detection of microRNAs in human urine, achieving a LOD of 1 nM [31].

**Figure 4:**
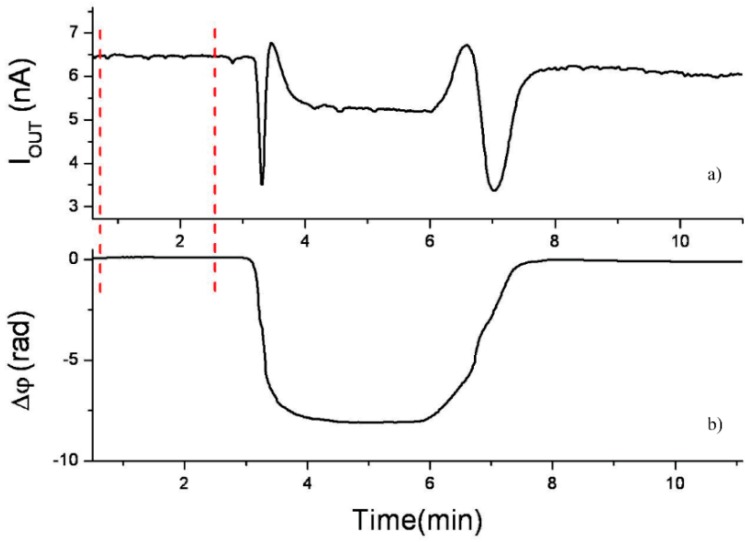
Response in a MZI sensor. (**a**) Standard interferometric signal; (**b**) Linear readout using a modulation system. Reprinted from [36] with permission of the Optical Society of America.

One of the main drawbacks of MZI biosensors is the complex interferometric nature of the output signal (see Figure 4a). As Equation (1) shows, the sinusoidal dependence of the intensity introduces ambiguities for a clear evaluation of the sensor response. A modulation system can help to translate the standard interferometric signal into an unambiguous linear phase evaluation, as Figure 4b shows. Magneto-optical [32], electro-optical [33], thermo-optical [34] or liquid crystal [35] modulation approaches have been proposed, but all these schemes rely on complex fabrication processes and bulk electronic equipment. More recently, to improve the integration and cost of the device, a new all-optical wavelength modulation system has been proposed. In this scheme the emission wavelength of a low-cost commercial laser diode was modulated ±2 nm by controlling its output power. After a Fast Fourier Transform deconvolution, the linear response of the biosensor is obtained. The all-optical modulated MZI biosensor was demonstrated through the linear sensing of the immunoreaction of the pair hGH/anti-hGH [36].

Other novel coherent detection scheme proposed to unambiguously extract the phase signal from a MZI modified with a three waveguides output coupler, uses a 2 × 3 multimode interferometer (MMI), and a processing of the output power with coherent receiver techniques. The complete amplitude and phase responses of the sensor can be recovered, avoiding the regions with zero sensitivity and without requiring any wavelength tuning laser. Until now, this coherent MZI sensor has not been applied as a biosensor [37].

Another alternative is to employ white-light to solve the limitations of single-wavelength sensors [38]. The use of multiple wavelengths can avoid the ambiguity and signal fading since each wavelength is affected in a different way by a refractive index variation. In an advanced configuration, Discrete Fourier Transform deconvolution was applied to direct and unambiguously retrieve the phase information from the sinusoidal transmission curves for each polarization [39]. This modulation has been implemented in an all-silicon monolithic MZI biosensor [40].

The Young Interferometer (YI) has a configuration similar to a MZI, but in YI the reference and sensing arms are not recombined using a Y-junction. In this case, both arms are out-coupled individually and the interference pattern is generated off-chip, which can be projected on a screen or CCD camera. The phase difference, *Δϕ(t)*, between the two interfering beams is given by:

(3) $$\Delta \varphi \left(t\right)=\frac{2\pi }{\lambda }\left[\frac{d\cdot x}{f}-\left(n_{eff}^{s}-n_{eff}^{r}\right)L\right]$$

where *d* is the distance between the two arms, *x* the position of the interference pattern on the screen, *f* the distance between the sensor output and the camera and $n_{eff}^{s}$ and $n_{eff}^{r}$ are the effective refractive indices of the guided modes in the sensing and reference arms, respectively.

Many advances have been achieved since the first integrated YI was proposed in 1994 [41]. The first application of this structure as a biosensor, using Si-technology, was reported in 2000 [42], with a detection limit of 9 × 10^−8^ RIU. Using this device, analytes in human plasma were evaluated [43]. A multi-analytical YI sensor of four branches (one of them as reference) was proposed in [44] showing the detection of herpes simplex virus type 1 (HSV-1), estimating that the detection limit can approach even the level of a single HSV-1 particle binding [45]. Recent advances have shown, theoretically, the possibility of applying the YI for analyte size-selective detection by launching multiple wavelengths, which allow discriminating between refractive index changes from different locations [46].

Other YI configurations, designed with two slab waveguides, have been developed [47]. This structure employs Ta_2_O_5_ as waveguide core, and is the most sensitive biosensor reported until now, with 9 × 10^−9^ RIU and 0.013 pg/mm^2^ for bulk and surface sensitivity, respectively [48]. Another version of YI is the Dual Polarization Interferometry (DPI) sensor [49], which is composed by five layers, forming two slab waveguides. One slab waveguide is used as reference and the other senses the changes occurring on its surface. DPI uses TM and TE polarization, allowing determination of the thickness and the refractive index of a film adsorbed on the sensor surface, by simultaneously measuring both polarizations. DPI is scarcely improved nowadays.

Recently, polymer-based YI have also been developed in order to improve the cost-efficiency and mass production [50,51], but the instability of the polymers, especially when they are in contact with a buffer solution, produces changes in the refractive index of the polymer due to water absorption, preventing their use for biosensing. Due to the continuous progress in polymer material research, advances in polymer-based IO biosensor are expected to appear in the near future.

**Figure 5:**
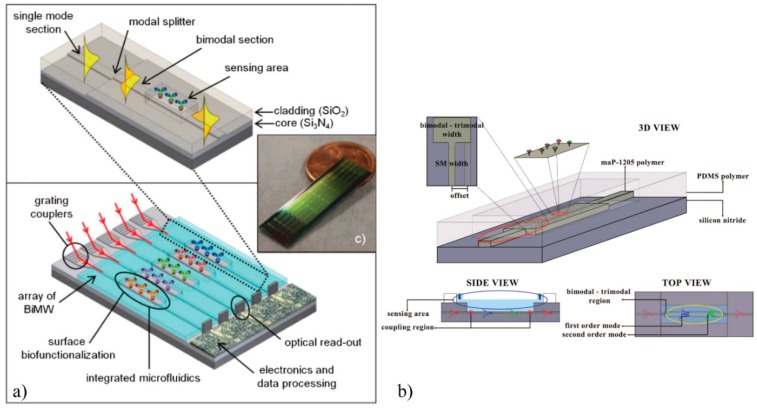
(**a**) LOC device based on BiMW, reprinted from [53] with permission of the Royal Society of Chemistry (**b**) Trimodal waveguide, reprinted from [55] with permission of the Optical Society of America.

In the last few years, a new device based on a common path waveguide interferometer has been described, the so-called BiModal Waveguide (BiMW) Interferometer. In this structure, the light is coupled in a straight Si_3_N_4_ single mode rib waveguide and after a certain distance, using a step-junction, two transversal modes with the same polarization are excited. A sensing window is open over the bimodal section. The interference between both guided modes is collected by a two-sectional photodetector at the end of the device. The sensitivity level of the BiMW is comparable to other integrated interferometers with LODs of 2.5 × 10^−7^ RIU in bulk [52]. A LOC with BiMW has been proposed, integrating grating couplers and a SU-8 microfluidic [53] (see Figure 5a). To solve the ambiguity problem in BiMW, the same all-optical phase modulation method described above was implemented [54].

In order to obtain a low-cost device for common path waveguide interferometers, a trimodal polymer-based biosensor has been recently proposed [55] (see Figure 5b). Because the penetration depth of the evanescent field of the second order mode is greater than the first order mode, the sensitivity of this structure can be comparable to a bimodal waveguide interferometer of similar characteristics.

Another interferometric sensor recently described is a single-channel MZI which employs two-lateral-modes fabricated in a silicon-on-insulator platform. A bulk and surface sensitivity of 461.6 π/RIU and 1.135 π/ng·mm^−2^, respectively, have been reported. The biosensor capability was verified by evaluating biotin–streptavidin interactions [56].

### 2.2. Ring Resonators-Based Biosensors

**Figure 6:**
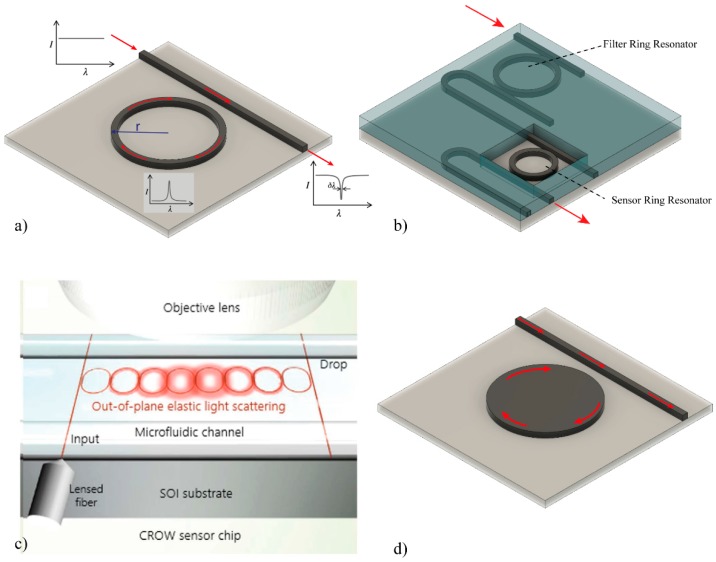
Ring resonator biosensors. (**a**) Conventional ring resonator; (**b**) two cascade ring resonators based on the Vernier-effect, which is redrawn based on [69]; (**c**) coupled-resonator optical-waveguide (CROW) [72] with permission of the Nature Publishing Group, and (**d**) micro-disk resonator [75].

Ring resonator biosensors have shown great potential, because they afford highly compact devices. In this structure, the light propagates through a straight waveguide and it is coupled into a ring waveguide where the light propagates in the form of whispering gallery modes, generating a resonance at a selected frequency (see Figure 6a). The wave will keep resonating inside the circular ring until adsorption and dissipation phenomena end up diminishing the energy resonating. The Q factor is a dimensionless indication of the efficiency of the resonator by relating the stored energy to the dissipated energy. A good resonator could reach Q factors as high as 10^4^, but in photonics, Q factors as high as 10^10^ have been reported. The effective length, *L_eff_*, of the device is directly related to the quality factor, *Q*, of the ring resonator as follows:

(4) $$L_{eff}=Q\frac{\lambda }{2\pi n}$$

where *n* is the resonator refractive index and the resonance wavelength, *λ*, is given by:

(5) $$\lambda =2\pi r\frac{n_{eff}}{m}$$

where *r* is the ring radius, *n_eff_* is the waveguide effective refractive index and *m* an integer number.

When a ring resonator is used as a biosensor, the surface of the ring must be uncovered, allowing the evanescent wave interaction between the waveguide and the external environment, detecting any refractive index variation at the surface. Many interesting publications have dealt with ring resonators made out of several materials such as: Si_3_N_4_ [57], polymer [58], Hydex [59], Si [60,61], achieving sensitivities in general in the range of 10^−6^ RIU [62]. One of the main advantages of ring resonator devices is the possibility of miniaturization as compared with other optical biosensors. Therefore, many advances in the integration of these structures have been presented, such as multiplexed sensing [63] or enhancements in microfluidic, in-coupling and readout [64,65,66,67]. Ring resonators arrays functionalized with clinically relevant biomarkers [63] have also been used to develop LOC devices [68].

In order to improve the LOD of these devices, different configurations of the ring resonator have been published, such as two cascade ring resonators based on the Vernier-effect (see Figure 6b). The first experimental results with this configuration in SOI were published in 2010, achieving a sensitivity of 2169 nm/RIU and a LOD of 8.3 × 10^−6^ RIU [69]. In addition, a double-ring resonators in cascaded able to simultaneous detect multiple species, and increasing the sensitivity until 24,300 nm/RIU, were proposed [70]. Integrating a microfluidic channels, this configuration was used as biosensor by covalently immobilizing streptavidin and measuring the binding capacity of biotylinated-hIgG, exhibiting a detection limit down to 7.1 μg/mL [71].

However, a main drawback of RR devices is the need to employ a tunable laser to excite the cavity resonance. A recently proposed solution is the use of a coupled-resonator optical-waveguide (CROW) (see Figure 6c) in SOI, which only requires a fixed wavelength to excite the CROW [72]. For that, the intensity of the light-scattering of each ring was captured, and an intensity pattern was generated which depends on the refractive index change upon the CROW. Until now, no biosensor application has been published.

An alternative to the resonant ring sensor is the resonant disk [73,74] (see Figure 6d). This device operates by monitoring the change in the transfer characteristics of the resonator disc when the analytes are deposited on the active area. Comparing with ring resonators, they afford higher sensitivities because the light-wave interacts many times with each analyte due to the resonance recirculation of light within the structure of the micro-disc. A biosensor capable of multiplexed interrogation of biological samples using micro-disk resonators has been evaluated for streptavidin detection by using a sandwich immunoassay and a biotin-conjugated BSA for signal amplification [75].

### 2.3. Photonic Crystals-Based Biosensors

**Figure 7:**
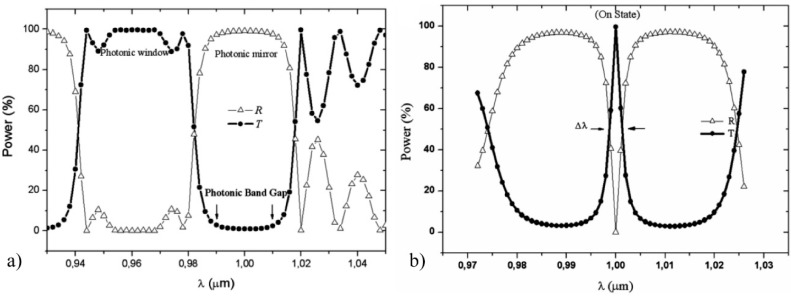
Transmitted (T) and reflected (R) power in a Photonic Crystal transducer [76], with permission of The Electromagnetics Academy. (**a**) Without any defect; (**b**) Applying a defect in the structure.

A photonic crystal (PC) is composed by periodic nanostructures with different refractive index materials, which affect the propagation of electromagnetic waves. These periodic structures generate a range of wavelengths, which are not propagated in the PC waveguide, called photonic bandgap (see Figure 7a). If a defect is introduced in the structure, the photonic bandgap will be modified as Figure 7b shows, and a peak centred on the frequency *f_0_* and bandwidth *Δf*, can be observed in the transmission or reflection spectrum of the PC waveguide. One of the most important characteristics of a PC is the Quality factor (Q-factor), which is defined as:

(6) $$Q=\frac{f_{0}}{\Delta f}$$

PC can be exploited for sensing [76] taking into account the Q-factor, the periodicity of the structure and the change of the refractive index between the dielectric materials.

In the early 21th century, the first PC biosensors were developed, using a polymer grating coated with a high refractive index layer (TIO_2_) [77,78,79]. In this case, white light impacted on the PC sensor and the reflected light was collected. Biomolecular interactions produced on the sensor surface generate a shift in the collected light. Some advances with this kind of PC biosensors have been published [80,81,82,83]. Different materials and layouts have been used as core. For example, SOI-based 2D PC nanocavity biosensors were developed, showing a minimum mass coverage of only 2.5 fg [84]. To allow a multiplexed evaluation, an array of three nanocavities (with different characteristics) coupled to a PC waveguide was developed, but due to the low Q-factor of the cavities (around 400), a non-competitive LOD in bulk of about 10^−2^ RIU and a surface sensitivity of 1 ng/mm^2^ for the detection of IgG was obtained [85].

**Figure 8:**
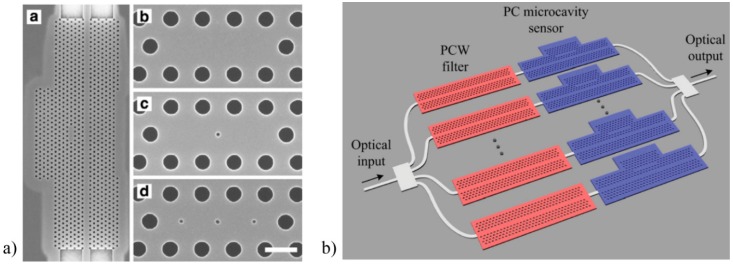
Advances in PCs biosensors. (**a**) Multiple hole defects (MHDs) [88], with permission of the Optical Society of America; (**b**) Transmission bandpass combined with PC sensor, with permission of the AIP Publishing LLC [90].

In order to improve the sensitivity of PC biosensors, the dimensions and distribution of the defects were investigated, such as three missing holes (*L3*), tuned radius of a single hole (*H1-r*) or width modulated cavity (WMC), obtaining a LOD 500 pg/mm^2^ [86,87]. Another alternative to increase the LOD, without degrading the Q-factor, is to introduce multiple hole defects (MHDs), which increase the surface area available for label-free detection by directly placing holes with diameter much smaller than the lattice constant as Figure 8a shows [88].

Advances in the detection of a biomarker from lung cancer cell lysates in complex mixtures using multiplexed SOI-based PC biosensor was demonstrated, achieving a sensitivity down to 2 cells/μL [89]. More recently, and in order to improve the integration of a multiplexed device, an additional PC waveguide filter was connected in series with each PC microcavity sensor. Thus, a transmission bandpass was created, which contained the resonances of the PC for sensing purpose and allowed use of a single input and a single output port (see Figure 8b) [90].

One-dimensional photonic crystals (1 DPC), using a periodic multilayer structure of SiO_2_-Ta_2_O_5_ were developed as biosensor. Using this structure, the covalent binding of a protein on COOH-rich polymeric film was evaluated, through the emissive behaviour of the photonic structure when the polymeric layer is impregnated with Cy3 dye [91]. Furthermore, 1 DPC has been used to measure biotin molecules binding to a streptavidin monolayer as well as the association and dissociation kinetics of immunoglobulin G proteins [92].

Slot waveguides have also been combined in PC [93], resulting in more sensitive devices than conventional PC sensors, reaching Q-factors of up to 50,000, a sensitivity of 1500 nm/RIU and a LOD of 7 × 10^−6^ RIU [94].

### 2.4. Optical Biosensors Comparison

Table 1 summarizes the configurations and results obtained so far in the field of silicon-based optical biosensors, described in the previous sections. Table 1 includes a comparison of the waveguide material and structure of the different sensors and the limit of detection, both in bulk (minimum change of refractive index unit) and in surface sensitivity (minimum detectable amount of material per mm^2^).

## 3. Prospects of Near Future Commercialization

It is clear that the challenges faced by each type of biosensor will depend on the targeted application. However, we can say that all integrated optical biosensors share common problems when developing a complete LOC device: the light source, the light incoupling and the read-out process, the delivery and disposal of the sample, the biofunctionalization of the sensor surface, the necessity to ensure an adequate time period of useful life as well as handling limitations (*i.e.*, storing and temperature and humidity valid ranges, *etc.*). These are the main reasons that have slowed the market boom in optical biosensors. Even though, there are a few companies already commercializing biosensors based on silicon-IO technologies, which are listed in Table 2.

Axela (Toronto, ON, Canada) has developed the dotLab mX System^®^ with proprietary Diffractive Optics Technology (dot^®^). The biosensor chip is built in a plastic cartridge including the sample delivery system and the biofunctionalization of the gratings with protein, DNA and/or RNA. They developed the software, methods for analysis, chips, sensors and reagents. Basically, they measure the diffraction angle shift in a previously bio-functionalized grating due to a change in the surface biolayer thickness by a specific binding event.

Corning (Corning, New York, NY, USA) commercializes a label free detection equipment based on Resonant Waveguide Grating Biosensor System named Corning^®^ Epic^®^. They use microarrays with up to 384 wells for multiplexed detection. Each microplate is composed of a Nb_2_O_5_ (n = 2.36) thin film biofunctionalized grating over a glass (n = 1.5) substrate. The shift in the reflected angle is related to the changes in the refractive index in the surface of the gratings.

Genalyte (San Diego, CA, USA) sells the Maverick^®^. This detection system is based in SOI ring resonators technology being able to process an assay in 15 min time. Each chip contains 128 separated ring resonators for multiplexed detection. The only drawback is the bulky size of the apparatus [95].

OWLS (Budapest, Hungary), the Hungarian division of Microvacuum Inc. (Budapest, Hungary), commercializes the OWLS210^®^ which uses DPI to relate the angle shift of red light coupled by gratings into a Si_x_Ti_(1−x)_O_2_ (where x = 0.25 ± 0.05) waveguide to the changes of the refractive index on the surface of the gratings. These alterations of the refractive index are related to the binding events of biological analytes [96].

As future perspectives, the incursion of large companies like GE, Google or Apple into the health and wearable devices market could drive the development of portable bio(chemical) sensing devices [97,98,99,100,101]. The inclusion of the mayor players of the smartphone operating systems industries is a clear indication of the growing role of health detection and monitoring systems among society’s demands. Smartphones nowadays incorporate high processing capabilities, network connectivity, a high range of physical sensors (gyroscopes, CCDs, touch screen, capacitive sensing, sound), several kinds of sources (LEDs, speakers, electrical power, *etc.*). They possess an intrinsic ubiquity: 94% of the world population are cell phone subscribers, 70% in developing countries; which make them ideal candidates to build immunoassays, microscopy, optical biosensors, lateral flow assays, flow cytometry and colorimetric detection in both urban and rural areas [102]. There is a clear dominance of the optical biosensors due to constant improvements in camera’s performance and the numerous LOC solutions which can be adapted to several types of phones [103].

In other hand, there are prizes such as the Qualcomm Tricorder Xprize ($10 million to build an automatic non-invasive portable device to detect a dozen medical conditions), the EU “Horizon Prize for better use of antibiotics” (€1 million to develop a device to rapidly differentiate between patients with respiratory infections that require antibiotics and patients that can be treated without them) or the UK Longitude Prize (£10 million to develop a cost effective, rapid, precise and user friendly test to differentiate between antibiotic resistant bacterial strains). These are examples of efforts made by large companies and governments to stimulate the research and development in this area attracting interdisciplinary teams of experts across the world. In addition, a number of open source community laboratories have emerged across the globe with the aim of developing more accessible, low-cost and user-friendly biosensors [104]. All of these efforts made by large companies, governments and think tanks indicate growing recognition of the important role that biosensors will play in improving the health of society. The technology is advancing frenetically and it seems that medical diagnostic technologies based on silicon-based optical biosensors have a bright future.

## Figures and Tables

**Table 1:** Comparison of optical biosensors.

Device	Mass Detection Limit (pg/mm^2^)	RI detection Limit (RIU)	Waveguide Structure	Waveguide Material	Reference
**Interferometric**					
**MZI**	0.06	1 × 10^−7^	Rib	Si_3_N_4_	[21]
****	0.25	ND*	Ridge	SOI	[23]
**Young**	0.75	9 × 10^−8^	Rib	Si_x_O_y_N_z_	[42]
****	0.013	9 × 10^−9^	Slab	Ta_2_O_5_	[48]
**Bimodal WG**	0.05	2.5 × 10^−7^	Rib	Si_3_N_4_	[52]
**Ring resonator**	1.5	7.6 × 10^−7^	Ridge	SOI	[63]
****	ND	8.3 × 10^−6^	Ridge	SOI	[69]
**Photonic crystal**	0.42	3.4 × 10^−5^	2D	Si_3_N_4_	[77]
****	ND	7 × 10^−6^	Slot	SOI	[94]

* ND not determined.

**Table 2:** Commercial solutions of biosensors based on integrated optics.

Company	Instrument	Technology	Webpage
**Axela**	DotLab	Optical grating	www.axelabiosensors.com
**Corning**	EPIC System	Resonant Gratings	www.corning.com
**Genalyte**	Maverick	Ring Resonator	www.genalyte.com
**OWLS**	OWLS210	DPI	www.owls-sensors.com

## References

[1] Domínguez E., Narváez A. (2005). Biosensors and Modern Biospecific Analytical Techniques.

[2] Estevez M.C., Alvarez M., Lechuga L.M. (2012). Integrated optical devices for lab-on-a-chip biosensing applications. Laser Photon. Rev..

[3] Fan X., White I.M., Shopova S.I., Zhu H., Suter J.D., Sun Y. (2008). Sensitive optical biosensors for unlabeled targets: A review. Anal. Chim Acta.

[4] Fan X., White I.M. (2011). Optofluidic Microsystems for Chemical and Biological Analysis. Nat. Photonics.

[5] Lambeck P.V. (2006). Integrated optical sensors for the chemical domain. Meas. Sci. Technol..

[6] Kussrow A., Enders C.S., Bornhop D.J. (2012). Interferometric Methods for Label-Free Molecular Interaction Studies. Anal. Chem..

[7] Jokerst J.V., Jacobson J.W., Bhagwandin B.D., Floriano P.N., Christodoulides N., Mcdevitt J.T. (2010). Programmable Nano-Bio-Chip Sensors : Analytical Meets Clinical. Anal. Chem..

[8] Duval D., Lechuga L.M. (2013). Breakthroughs in Photonics 2012: 2012 Breakthroughs in Lab-on-a-Chip and Optical Biosensors. IEEE Photonics J..

[9] Duval D., Lechuga L.M., Andrews D.L. (2015). Optical Waveguide Biosensors. Photonics: Scientific Foundations, Technology and Applications, IV.

[10] De Vos K., Girones J., Popelka S., Schacht E., Baets R., Bienstman P. (2009). SOI optical microring resonator with poly(ethylene glycol) polymer brush for label-free biosensor applications. Biosens. Bioelectron..

[11] Ram M.K., Bertoncello P., Ding H., Paddeu S., Nicolini C. (2001). Cholesterol biosensors prepared by layer-by-layer technique. Biosens. Bioelectron..

[12] Cheng Z., Tsang H.K., Xu K., Shi Z. (2013). Spectral hole burning in silicon waveguides with a graphene layer on top. Opt. Lett..

[13] Rich R.L., Myszka D.G. (2011). Survey of the 2009 commercial optical biosensor literature. J. Mol. Recognit..

[14] Kozma P., Kehl F., Ehrentreich-Förster E., Stamm C., Bier F.F. (2014). Integrated planar optical waveguide interferometer biosensors: A comparative review. Biosens. Bioelectron..

[15] Heideman R.G., Kooyman R.P.H., Greve J. (1991). Development of an optical waveguide interferometric immunosensor. Sens. Actuators B Chem..

[16] Lechuga L.M., Lenferink A.T.M., Kooyman R.P.H., Greve J. (1995). Feasibility of evanescent wave interferometer immunosensors for pesticide detection: chemical aspects. Sens. Actuators B Chem..

[17] Schipper E.F., Brugman A.M., Dominguez C., Lechuga L.M., Kooyman R.P.H., Greve J. (1997). The realization of an integrated Mach-Zehnder waveguide immunosensor in silicon technology. Sens. Actuators B Chem..

[18] Prieto F., Sepúlveda B., Calle A., Llobera A., Domínguez C., Lechuga L.M. (2003). Integrated Mach-Zehnder interferometer based on ARROW structures for biosensor applications. Sens. Actuators B Chem..

[19] Prieto F., Lechuga L.M., Calle A., Llobera A., Domínguez C. (2001). Optimized silicon antiresonant reflecting optical waveguides for sensing applications. J. Light. Technol..

[20] Prieto F., Sep lveda B., Calle A., Llobera A., Domínguez C., Abad A., Montoya A., Lechuga L.M. (2003). An integrated optical interferometric nanodevice based on silicon technology for biosensor applications. Nanotechnology.

[21] Zinoviev K., Carrascosa L.G., Del Río J.S., Sepúlveda B., Domínguez C., Lechuga L.M. (2008). Silicon photonic biosensors for lab-on-a-chip applications. Adv. Opt. Technol..

[22] Densmore A., Xu D.X., Waldron P., Janz S., Cheben P., Lapointe J., Delâge A., Lamontagne B., Schmid J.H., Post E. (2006). A silicon-on-insulator photonic wire based evanescent field sensor. IEEE Photonics Technol. Lett..

[23] Densmore A., Vachon M., Xu D.-X., Janz S., Ma R., Li Y.-H., Lopinski G., Delâge A., Lapointe J., Luebbert C.C. (2009). Silicon photonic wire biosensor array for multiplexed real-time and label-free molecular detection. Opt. Lett..

[24] Mathesz A., Fábián L., Valkai S., Alexandre D., Marques P.V.S., Ormos P., Wolff E.K., Dér A. (2013). High-speed integrated optical logic based on the protein bacteriorhodopsin. Biosens. Bioelectron..

[25] Crespi A., Gu Y., Ngamsom B., Hoekstra H.J.W.M., Dongre C., Pollnau M., Ramponi R., van den Vlekkert H.H., Watts P., Cerullo G. (2010). Three-dimensional Mach-Zehnder interferometer in a microfluidic chip for spatially-resolved label-free detection. Lab Chip.

[26] Choo S.J., Lee B.-C., Lee S.-M., Park J.H., Shin H.-J. (2009). Optimization of silicon oxynitrides by plasma-enhanced chemical vapor deposition for an interferometric biosensor. J. Micromech. Microeng..

[27] Choo S.J., Kim J., Lee K.W., Lee D.H., Shin H.J., Park J.H. (2014). An integrated Mach-Zehnder interferometric biosensor with a silicon oxynitride waveguide by plasma-enhanced chemical vapor deposition. Curr. Appl. Phys..

[28] Duval D., Osmond J., Dante S., Domínguez C., Lechuga L.M. (2013). Grating couplers integrated on Mach-Zehnder interferometric biosensors operating in the visible range. IEEE Photonics J..

[29] Almeida V.R., Xu Q., Barrios C.A., Lipson M. (2004). Guiding and confining light in void nanostructure. Opt. Lett..

[30] Liu Q., Tu X., Kim K.W., Kee J.S., Shin Y., Han K., Yoon Y.J., Lo G.Q., Park M.K. (2013). Highly sensitive Mach-Zehnder interferometer biosensor based on silicon nitride slot waveguide. Sens. Actuators B Chem..

[31] Liu Q., Shin Y., Kee J.S., Kim K.W., Rafei S.R.M., Perera A.P., Tu X., Lo G.-Q., Ricci E., Colombel M. (2015). Mach–Zehnder interferometer (MZI) point-of-care system for rapid multiplexed detection of microRNAs in human urine specimens. Biosens. Bioelectron..

[32] Sepúlveda B., Armelles G., Lechuga L.M. (2007). Magneto-optical phase modulation in integrated Mach-Zehnder interferometric sensors. Sens. Actuators A Phys..

[33] Lambeck P.V. (1999). Remote opto-chemical sensing with extreme sensitivity: Design, fabrication and performance of a pigtailed integrated optical phase-modulated Mach-Zehnder interferometer system. Sens. Actuators B Chem..

[34] Dumais P., Callender C.L., Noad J.P., Ledderhof C.J. (2008). Integrated optical sensor using a liquid-core waveguide in a Mach-Zehnder interferometer. Opt. Express.

[35] Kozma P., Hamori A., Cottier K., Kurunczi S., Horvath R. (2009). Grating coupled interferometry for optical sensing. Appl. Phys. B Lasers Opt..

[36] Dante S., Duval D., Sepúlveda B., González-Guerrero A.B., Sendra J.R., Lechuga L.M. (2012). All-optical phase modulation for integrated interferometric biosensors. Opt. Express.

[37] Halir R., Vivien L., Le Roux X., Xu D.X., Cheben P. (2013). Direct and sensitive phase readout for integrated waveguide sensors. IEEE Photonics J..

[38] Kitsara M., Misiakos K., Raptis I., Makarona E. (2010). Integrated optical frequency-resolved Mach-Zehnder interferometers for label-free affinity sensing. Opt. Express.

[39] Misiakos K., Raptis I., Salapatas A. (2014). Broad-band Mach-Zehnder interferometers as high performance refractive index sensors: Theory and monolithic implementation. Opt. Express.

[40] Misiakos K., Raptis I., Makarona E., Botsialas A., Salapatas A., Oikonomou P., Psarouli A., Petrou P.S., Kakabakos S.E., Tukkiniemi K., Sopanen M., Jobst G. (2014). All-silicon monolithic Mach-Zehnder interferometer as a refractive index and bio-chemical sensor. Opt. Express.

[41] Brandenburg A., Henninger R. (1994). Integrated optical Young interferometer. Appl. Opt..

[42] Brandenburg A., Krauter R., Künzel C., Stefan M., Schulte H. (2000). Interferometric sensor for detection of surface-bound bioreactions. Appl. Opt..

[43] Brynda E., Houska M., Brandenburg A., Wikerstål A. (2002). Optical biosensors for real-time measurement of analytes in blood plasma. Biosens. Bioelectron..

[44] Ymeti A., Kanger J.S., Greve J., Lambeck P.V., Wijn R., Heideman R.G. (2003). Realization of a multichannel integrated Young interferometer chemical sensor. Appl. Opt..

[45] Ymeti A., Greve J., Lambeck P.V., Wink T., Van Novell S.W.F.M., Beumer T.A.M., Wijn R.R., Heideman R.G., Subramaniam V., Kanger J.S. (2007). Fast, ultrasensitive virus detection using a young interferometer sensor. Nano Lett..

[46] Mulder H.K.P., Ymeti A., Subramaniam V., Kanger J.S. (2012). Size-selective detection in integrated optical interferometric biosensors. Opt. Express.

[47] Schmitt K., Schirmer B., Brandenburg A. Label-free detection of biomolecules by waveguide interferometry. Proceedings of the 17th International Conference on Optical Fibre Sensors.

[48] Schmitt K., Schirmer B., Hoffmann C., Brandenburg A., Meyrueis P. (2007). Interferometric biosensor based on planar optical waveguide sensor chips for label-free detection of surface bound bioreactions. Biosens. Bioelectron..

[49] Cross G.H., Reeves A.A., Brand S., Popplewell J.F., Peel L.L., Swann M.J., Freeman N.J. (2003). A new quantitative optical biosensor for protein characterisation. Biosens. Bioelectron..

[50] Wang M., Hiltunen J., Liedert C., Pearce S., Charlton M., Hakalahti L., Karioja P., Myllylä R. (2012). Highly sensitive biosensor based on UV-imprinted layered polymeric–inorganic composite waveguides. Opt. Express.

[51] Wang M., Uusitalo S., Liedert C., Hiltunen J., Hakalahti L., Myllylä R. (2012). Polymeric dual-slab waveguide interferometer for biochemical sensing applications. Appl. Opt..

[52] Zinoviev K.E., González-Guerrero A.B., Domínguez C., Lechuga L.M. (2011). Integrated bimodal waveguide interferometric biosensor for label-free analysis. J. Light. Technol..

[53] Duval D., González-Guerrero A.B., Dante S., Osmond J., Monge R., Fernández L.J., Zinoviev K.E., Domínguez C., Lechuga L.M. (2012). Nanophotonic lab-on-a-chip platforms including novel bimodal interferometers, microfluidics and grating couplers. Lab Chip.

[54] Dante S., Duval D., Fariña D., González-Guerrero A.B., Lechuga L.M. (2015). Linear readout of integrated interferometric biosensors using a periodic wavelength modulation. Laser Photon. Rev..

[55] Ramirez J.C., Lechuga L.M., Gabrielli L.H., Hernandez-Figueroa H.E. (2015). Study of a low-cost trimodal polymer waveguide for interferometric optical biosensors. Opt. Express.

[56] Liu Q., Kim K.W., Gu Z., Kee J.S., Park M.K. (2014). Single-channel Mach-Zehnder interferometric biochemical sensor based on two-lateral-mode spiral waveguide. Opt. Express.

[57] Sohlstrom H., Oberg M. Refractive Index Measurement Using Integrated Ring Resonators. Proceedings of the 8th European Conference on Integrated Optics ECIO.

[58] Chao C.Y., Fung W., Guo L.J. (2006). Polymer microring resonators for biochemical sensing applications. IEEE J. Sel. Top. Quantum Electron..

[59] Yalçin A., Popat K.C., Aldridge J.C., Desai T.A., Hryniewicz J., Chbouki N., Little B.E., King O., Van V., Chu S. (2006). Optical sensing of biomolecules using microring resonators. IEEE J. Sel. Top. Quantum Electron..

[60] De Vos K., Bartolozzi I., Schacht E., Bienstman P., Baets R. (2007). Silicon-on-Insulator microring resonator for sensitive and label-free biosensing. Opt. Express.

[61] Park M.K., Kee J.S., Quah J.Y., Netto V., Song J., Fang Q., La Fosse E.M., Lo G.Q. (2013). Label-free aptamer sensor based on silicon microring resonators. Sens. Actuators B Chem..

[62] Washburn A.L., Gunn L.C., Bailey R.C. (2009). Label-free quantitation of a cancer biomarker in complex media using silicon photonic microring resonators. Anal. Chem..

[63] Iqbal M., Gleeson M.A., Spaugh B., Tybor F., Gunn W.G., Hochberg M., Baehr-Jones T., Bailey R.C., Gunn L.C. (2010). Label-free biosensor arrays based on silicon ring resonators and high-speed optical scanning instrumentation. IEEE J. Sel. Top. Quantum Electron..

[64] Densmore A., Xu D.X., Cheben P., Vachon M., Janz S., Ma R., Bedard D., Li Y., Lopinski G., Delâge A. Integration of vertical grating couplers and microfluidic channels with silicon photonic wire biosensor arrays. Proceedings of the 2010 IEEE Sensors.

[65] Xu D.-X., Vachon M., Densmore A., Ma R., Delâge A., Janz S., Lapointe J., Li Y., Lopinski G., Zhang D. (2010). Label-free biosensor array based on silicon-on-insulator ring resonators addressed using a WDM approach. Opt. Lett..

[66] Xu D.-X., Vachon M., Densmore A., Ma R., Janz S., Delâge A., Lapointe J., Cheben P., Schmid J.H., Post E. (2010). Real-time cancellation of temperature induced resonance shifts in SOI wire waveguide ring resonator label-free biosensor arrays. Opt. Express.

[67] Atsumi Y., Xu D.-X., Delâge A., Schmid J.H., Vachon M., Cheben P., Janz S., Nishiyama N., Arai S. (2012). Simultaneous retrieval of fluidic refractive index and surface adsorbed molecular film thickness using silicon wire waveguide biosensors. Opt. Express.

[68] Barrios C.A. (2012). Integrated microring resonator sensor arrays for labs-on-chips. Anal. Bioanal. Chem..

[69] Claes T., Bogaerts W., Bienstman P. (2010). Experimental characterization of a silicon photonic biosensor consisting of two cascaded ring resonators based on the Vernier-effect and introduction of a curve fitting method for an improved detection limit. Opt. Express.

[70] Jiang X., Ye J., Zou J., Li M., He J.-J. (2013). Cascaded silicon-on-insulator double-ring sensors operating in high-sensitivity transverse-magnetic mode. Opt. Lett..

[71] Chen Y., Yu F., Yang C., Song J., Tang L., Li M., He J.-J. (2015). Label-free biosensing using cascaded double-microring resonators integrated with microfluidic channels. Opt. Commun..

[72] Wang J., Yao Z., Lei T., Poon A.W. (2014). Silicon coupled-resonator optical-waveguide-based biosensors using light-scattering pattern recognition with pixelized mode-field-intensity distributions. Sci. Rep..

[73] Boyd R.W., Heebner J.E. (2001). Sensitive disk resonator photonic biosensor. Appl. Opt..

[74] Krioukov E., Klunder D.J.W., Driessen A., Greve J., Otto C. (2002). Sensor based on an integrated optical microcavity. Opt. Lett..

[75] Grist S.M., Schmidt S.A., Flueckiger J., Donzella V., Shi W., Fard S.T., Kirk J.T., Ratner D.M., Cheung K.C., Chrostowski L. (2013). Silicon photonic micro-disk resonators for label-free biosensing. Opt. Express.

[76] Garcia J.R., Granda M.G., Gavela A.F., Presa S.A., Lastra M.R., Fernández S.F. (2012). Electromagnetic Waves Scattering at interfaces Between Dielectric Waveguides: A Review on Analysis and Applications. Prog. Electromagn. Res. B.

[77] Cunningham B., Li P., Lin B., Pepper J. (2002). Colorimetric resonant reflection as a direct biochemical assay technique. Sens. Actuators B Chem..

[78] Cunningham B., Lin B., Qiu J., Li P., Pepper J., Hugh B. (2002). A plastic colorimetric resonant optical biosensor for multiparallel detection of label-free biochemical interactions. Sens. Actuators B Chem..

[79] Cunningham B., Qiu J., Li P., Lin B. (2002). Enhancing the surface sensitivity of colorimetric resonant optical biosensors. Sens. Actuators B Chem..

[80] Chan L.L., Cunningham B.T., Li P.Y., Puff D. (2007). Self-referenced assay method for photonic crystal biosensors: Application to small molecule analytes. Sens. Actuators B Chem..

[81] Chan L.L., Gosangari S.L., Watkin K.L., Cunningham B.T. (2008). Label-free imaging of cancer cells using photonic crystal biosensors and application to cytotoxicity screening of a natural compound library. Sens. Actuators B Chem..

[82] Schudel B.R., Choi C.J., Cunningham B.T., Kenis P.J.A. (2009). Microfluidic chip for combinatorial mixing and screening of assays. Lab Chip.

[83] Choi C.J., Belobraydich A.R., Chan L.L., Mathias P.C., Cunningham B.T. (2010). Comparison of label-free biosensing in microplate, microfluidic, and spot-based affinity capture assays. Anal. Biochem..

[84] Lee M.R., Fauchet P.M. (2007). Two-dimensional silicon photonic crystal based biosensing platform for protein detection. Opt. Express.

[85] Pal S., Guillermain E., Sriram R., Miller B.L., Fauchet P.M. (2011). Silicon photonic crystal nanocavity-coupled waveguides for error-corrected optical biosensing. Biosens. Bioelectron..

[86] Dorfner D.F., Hürlimann T., Zabel T., Frandsen L.H., Abstreiter G., Finley J.J. (2008). Silicon photonic crystal nanostructures for refractive index sensing. Appl. Phys. Lett..

[87] Dorfner D., Zabel T., Hürlimann T., Hauke N., Frandsen L., Rant U., Abstreiter G., Finley J. (2009). Photonic crystal nanostructures for optical biosensing applications. Biosens. Bioelectron..

[88] Kang C., Phare C.T., Vlasov Y.A., Assefa S., Weiss S.M. (2010). Photonic crystal slab sensor with enhanced surface area. Opt. Express.

[89] Chakravarty S., Lai W.-C., Zou Y., Drabkin H.A., Gemmill R.M., Simon G.R., Chin S.H., Chen R.T. (2013). Multiplexed specific label-free detection of NCI-H358 lung cancer cell line lysates with silicon based photonic crystal microcavity biosensors. Biosens. Bioelectron..

[90] Yan H., Zou Y., Chakravarty S., Yang C.-J., Wang Z., Tang N., Fan D., Chen R.T. (2015). Silicon on-chip bandpass filters for the multiplexing of high sensitivity photonic crystal microcavity biosensors. Appl. Phys. Lett..

[91] Frascella F., Ricciardi S., Rivolo P., Moi V., Giorgis F., Descrovi E., Michelotti F., Munzert P., Danz N., Napione L. (2013). A fluorescent one-dimensional photonic crystal for label-free biosensing based on BLOCH surface waves. Sensors.

[92] Konopsky V.N., Karakouz T., Alieva E.V., Vicario C., Sekatskii S.K., Dietler G. (2013). Photonic Crystal Biosensor based on Optical Surface Waves. Sensors.

[93] Scullion M.G., Krauss T.F., di Falco A. (2013). Slotted photonic crystal sensors. Sensors.

[94] Di Falco A., O’Faolain L., Krauss T.F. (2009). Chemical sensing in slotted photonic crystal heterostructure cavities. Appl. Phys. Lett..

[95] Luchansky M.S., Bailey R.C. (2012). High-Q optical sensors for chemical and biological analysis. Anal. Chem..

[96] Vörös J., Ramsden J.J., Csúcs G., Szendro I., de Paul S.M., Textor M., Spencer N.D. (2002). Optical grating coupler biosensors. Biomaterials.

[97] Mühlsteff J. (2015). Wearable Sensors in Syncope Management. Med. Sci. Monit..

[98] Pantelopoulos A., Bourbakis N.G. (2010). A survey on wearable sensor-based systems for health monitoring and prognosis. IEEE Trans. Syst. Man Cybern. Part. C Appl. Rev..

[99] Patel S., Park H., Bonato P., Chan L., Rodgers M. (2012). A review of wearable sensors and systems with application in rehabilitation. J. Neuroeng. Rehabil..

[100] Fletcher R.R., Poh M.Z., Eydgahi H. Wearable sensors: Opportunities and challenges for low-cost health care. Proceedings of the 2010 Annual International Conference of the IEEE Engineering in Medicine and Biology Society.

[101] Tao W., Liu T., Zheng R., Feng H. (2012). Gait analysis using wearable sensors. Sensors.

[102] Vashist S., Schneider E., Luong J. (2014). Commercial Smartphone-Based Devices and Smart Applications for Personalized Healthcare Monitoring and Management. Diagnostics.

[103] Preechaburana P., Suska A., Filippini D. (2014). Biosensing with cell phones. Trends Biotechnol..

[104] Rowe A.A., Bonham A.J., White R.J., Zimmer M.P., Yadgar R.J., Hobza T.M., Honea J.W., Ben-Yaacov I., Plaxco K.W. (2011). Cheapstat: An open-source, “do-it-yourself” potentiostat for analytical and educational applications. PLoS ONE.

